# Dietary Intake according to Gender and Education: A Twenty-Year Trend in a Swiss Adult Population

**DOI:** 10.3390/nu7115481

**Published:** 2015-11-18

**Authors:** Pedro Marques-Vidal, Eirini Rousi, Fred Paccaud, Jean-Michel Gaspoz, Jean-Marc Theler, Murielle Bochud, Silvia Stringhini, Idris Guessous

**Affiliations:** 1 Department of Internal Medicine, Internal Medicine, Lausanne University Hospital (CHUV), Lausanne 1010, Switzerland; 2 Institute of Social and Preventive Medicine (IUMSP), Lausanne University Hospital, Lausanne 1011, Switzerland; Eirini.Rousi@unil.ch (E.R.); Fred.Paccaud@chuv.ch (F.P.); Murielle.Bochud@chuv.ch (M.B.); Silvia.Stringhini@chuv.ch (S.S.); Idris.Guessous@hcuge.ch (I.G.); 3 Unit of Population Epidemiology, Division of Primary Care Medicine, Department of Community Medicine, Primary Care and Emergency Medicine, Geneva University Hospitals, Geneva 1205, Switzerland; Jean-Michel.Gaspoz@hcuge.ch (J.-M.G.); Jean-Marc.Theler@hcuge.ch (J.-M.T.); 4 Department of Epidemiology, Rollins School of Public Health, Emory University, Atlanta, GA 30322, USA

**Keywords:** nutrients, trends, epidemiology, public health, adult, educational level, gender, population-based study, Switzerland

## Abstract

We assessed trends in dietary intake according to gender and education using repeated cross-sectional, population-based surveys conducted between 1993 and 2012 in Geneva, Switzerland (17,263 participants, 52.0 ± 10.6 years, 48% male). In 1993–1999, higher educated men had higher monounsaturated fatty acids (MUFA), carotene and vitamin D intakes than lower educated men, and the differences decreased in 2006–2012. In 1993–1999, higher educated women had higher fiber, iron, carotene, vitamin D and alcohol intakes than lower educated women, and the differences decreased in 2006–2012. Total energy, polyunsaturated fatty acids, retinol and alcohol intakes decreased, while mono/disaccharides, MUFA and carotene intake increased in both genders. Lower educated men had stronger decreases in saturated fatty acid (SFA) and calcium intakes than higher educated men: multivariate-adjusted slope and 95% confidence interval −0.11 (−0.15; −0.06) *vs.* −0.03 (−0.08; 0.02) g/day/year for SFA and −5.2 (−7.8; −2.7) *vs.* −1.03 (−3.8; 1.8) mg/day/year for calcium, *p* for interaction <0.05. Higher educated women had a greater decrease in iron intake than lower educated women: −0.03 (−0.04; −0.02) *vs.* −0.01 (−0.02; 0.00) mg/day/year, *p* for interaction = 0.002. We conclude that, in Switzerland, dietary intake evolved similarly between 1993 and 2012 in both educational groups. Educational differences present in 1993 persisted in 2012.

## 1. Introduction

Nutrition can influence the development and progression of various chronic diseases, such as cardiovascular disease (CVD), diabetes and cancer [1,2,3]. Socioeconomic status (SES) is a strong determinant of dietary intake, and several studies conducted in high-income countries have shown that individuals with a higher educational level and/or income consume higher-quality diets [4,5,6] than their counterparts.

Switzerland is a high-income European country characterized by a low mortality from CVD [7] and a relatively slow increase in body mass index (BMI) and obesity levels during the past decades [8,9]. In a previous study based on the Food and Agricultural Organization food balance sheets, some of us have shown that total energy intake (TEI) remained relatively stable in Switzerland while mono/disaccharides availability increased; considerable changes in vegetable oil use were also observed [10]. Still, these analyses were conducted on aggregated data and no study assessed long-term trends (*i.e.*, over a decade) in dietary intake in the Swiss population. Such trends are important for the monitoring of dietary intake and for the implementation and evaluation of public health interventions [11]. Trends and determinant analyses can also guide local level interventions and might improve their efficacy, given that the interventions can then be tailored. Previous studies conducted in the canton of Geneva showed that individuals of high SES had a healthier diet [12] and that the diet of individuals of low SES worsened between 1993 and 2000 [13], but no other study has been performed since.

Thus, we aimed to assess (a) 20-year (1993–2012) trends in dietary intakes according to gender and educational level and (b) the differences in nutrient intakes between educational groups in a Swiss adult population across the study period.

## 2. Experimental Section

### 2.1. Study Design and Sampling

The “Bus Santé” study is a cross-sectional, on-going population-based study designed to collect information on chronic disease risk factors in the canton of Geneva, Switzerland. The sampling methodology of the “Bus Santé” Geneva study has been reported previously [14]. Briefly, every year since 1993, a representative sample of non-institutionalized men and women aged 35 to 74 years is recruited. Eligible subjects are identified with a standardized procedure using a residential list established annually by the local government. Random sampling in age and sex-specific strata is proportional to the corresponding frequencies in the population. A person who is not reached after three mailings and seven phone calls is replaced using the same selection protocol as above, but people who are reached and refuse to participate are not replaced. Included participants are not eligible for future recruitments and surveys. Participation rates ranged from 50% to 66% throughout the 20 years of this study.

### 2.2. Data Collected

Health examinations are conducted throughout the year, from January to December, in two clinics and one mobile medical unit. Body weight and height were measured using standard procedures, and BMI (kg/m^2^) was calculated. Data for socio-demographic characteristics, smoking and educational history were collected using self-administered, standardized questionnaires. Trained collaborators perform the examinations, interview the participants and check the self-administered questionnaires for completion. Procedures are regularly reviewed and standardized across collaborators.

Smoking status (never smokers, ex-smokers, current smokers) was self-reported. Marital status was categorized as single, married/cohabiting, divorced and widowed. Country of birth was classified as Switzerland, and “Other”, which was the combination of all other countries. Education, measured as credentials of formal schooling, was used as a proxy for SES. Due to changes in coding during the study period, educational level attained was categorized into “high” (university) and “low” (lower than university).

### 2.3. Dietary Intake

Food intake was assessed by a semi-quantitative food frequency questionnaire (FFQ) developed and validated in the target population [15]. Data derived from this FFQ have recently contributed to worldwide analyses [16,17]. Each FFQ was checked for completion by a trained interviewer on the day of the visit to the clinic, leading to a completion rate of 100%. The FFQ provides information about consumption frequencies and serving sizes during the 4 previous weeks for 97 fresh and prepared food items organized in 12 different food groups (dietary supplements not accounted for). To our knowledge, there is no validated FFQ assessing annual dietary intake in Switzerland, and it has been shown that FFQs assessing dietary intake for shorter periods than one year have the same validity as FFQs assessing annual dietary intake [18]. Thus, the FFQ used in this study is the best possible option to assess dietary intake in the Swiss French speaking population. Consumption frequencies range from “less than once during the last weeks” to “2 or more times per day” and three serving sizes (smaller, equal or larger than a reference size) are provided. The validation study showed that the food items account for over 90% of energy, protein, fat, carbohydrate, alcohol, cholesterol, vitamin D and retinol intake; 85% of fiber, carotene and iron intake; and 62% of calcium intake [19]. The FFQ data were converted into daily energy, alcohol and nutrients coming from the different food items using the French CIQUAL (Centre d’Information sur la QUalité des ALiments) food composition table. Total vitamin A was calculated as the sum of retinol (animal sources) and carotene (plant sources) using the formula *retinol + carotene/12* and the results were expressed in μg of retinol activity equivalents (RAE). For the present analysis, absolute values of total nutrients were used.

### 2.4. Exclusion Criteria

Participants with missing data for education, age, weight, height, marital status, smoking habits and country of birth or nationality were excluded, as well as participants with extreme energy intakes (<850 or >4000 kcal/day) or implausible values for nutrient intake (*i.e.*, >400 g/day of protein or >100 g/day fiber or >6000 mg/day calcium) as performed previously [20,21].

### 2.5. Ethics Statement

The “Bus Santé” Geneva study was approved by the University of Geneva ethics committee and all study participants provided informed written consent to participate in the study. The study has been performed in accordance with the ethical standards laid down in the 1964 Declaration of Helsinki and its later amendments.

### 2.6. Statistical Analysis

Statistical analyses were performed using Stata version 13.1 for windows (Stata Corp, College Station, TX, USA). Descriptive results were expressed as number of participants (percentage) or as mean ± standard deviation. To facilitate reading, the whole study period was divided in three periods of comparable length: 1993–1999 (7 years), 2000–2005 (6 years) and 2006–2012 (7 years). No specific hypotheses were made regarding dietary trends within these periods.

All trend analyses were stratified by gender. As in previous studies [10,22], we assumed stable yearly changes throughout the entire study period. Trends (yearly changes) for dietary intakes were assessed by linear regression using yearly (not grouped) data; effect modification by educational level was assessed by introducing an interaction term between educational level and survey year. Two models were used: (a) unadjusted or TEI-adjusted for nutrients; and (b) multivariable adjusted for age, smoking status, country of birth (dichotomized into Switzerland yes/no) and marital status, with a further adjustment on TEI for nutrients. Adjusting for age, smoking and marital status was considered as necessary as results from a study conducted in nearby Lausanne showed that these factors are associated with dietary intake [23]. As there were over 30 different countries of birth, it was decided not to split them, as this would lead to small sample sizes.

Differences in dietary intakes between educational levels were computed for the initial (1993–1999) and final (2006–2012) years of the study. As for trends, two models were used: (a) age or TEI-adjusted for nutrients and (b) multivariable adjusted for age, smoking status, country of birth and marital status, with a further adjustment on TEI for nutrients. Results were expressed as adjusted differences (lower than university minus university) and 95% confidence intervals (CI). A negative difference meaning that diet (nutrient) intake is lower among non-university than university participants.

Sensitivity analysis was conducted by including participants previously excluded because of extreme energy intakes. Due to the number of tests performed, statistical significance was considered for a two-sided test, *p* < 0.001.

## 3. Results

### 3.1. Selection of Participants and Characteristics of the Final Sample

Of the initial 19,147 participants, 1880 (9.8%) were excluded. The reasons for exclusion are summarized in the Figure S1 and the characteristics of the included and excluded participants are summarized in Table S1. Excluded participants were younger, more frequently single or smokers and less frequently born in Switzerland than included ones. No differences were found regarding BMI or educational level.

The characteristics of the participants included in the analysis are summarized in Table 1 according to the three survey periods. Over the study period, the percentage of participants with university level increased; mean BMI increased; the percentage of divorced participants increased at the expense of the married and the percentage of participants born outside Switzerland increased.

**Table 1:** Characteristics of the participants of the “Bus Santé” study, Geneva, Switzerland, according to study period.

	1993–1999	2000–2005	2006–2012	*p*-Value
Sample size	6922	6034	4307	
Women (%)	3665 (53.0)	3175 (52.6)	2169 (50.4)	0.02
Age (years)	51.8 ± 10.4	52.1 ± 10.7	52.3 ± 10.9	0.08
BMI (kg/m^2^)	24.5 ± 3.9	24.8 ± 4.0	25.1 ± 4.1	<0.001
Marital status (%)				
Single	623 (9.0)	631 (10.5)	430 (10.0)	
Married/cohabitating	5173 (74.7)	4362 (72.3)	3140 (72.9)	<0.001
Divorced	848 (12.3)	806 (13.4)	629 (14.6)	
Widowed	278 (4.0)	235 (3.9)	108 (2.5)	
Education (%)				
University	2111 (30.5)	2105 (34.9)	1872 (43.5)	<0.001
Lower than university	4811 (69.5)	3929 (65.1)	2435 (56.5)	
Smoking status (%)				
Never Smoked	3070 (44.4)	2690 (44.6)	2015 (46.8)	0.005
Ex-Smoker	2202 (31.8)	1943 (32.2)	1393 (32.3)	
Current smoker	1650 (23.8)	1401 (23.2)	899 (20.9)	
Country of birth (%)				
Switzerland	3946 (57.0)	3300 (54.7)	2252 (52.3)	<0.001
Other	2976 (43.0)	2734 (45.3)	2055 (47.7)	

Results are expressed as number of participants (percentage) or mean ± standard deviation. BMI, body mass index. Statistical comparisons by chi-square or ANOVA.

### 3.2. Trends in Energy and Nutrient Intake

TEI and nutrient intake according to gender and study period are summarized in Table S2 (unadjusted or energy-adjusted for nutrients) and in Table 2 (multivariable adjusted). In both genders, total energy, SFA, PUFA, calcium, vitamin A, retinol and alcohol intake decreased, while MUFA and carotene intake increased (Table 2). An increase in mono/disaccharide and total fiber intake was found in men only and a decrease in iron intake was found in women only (Table 2). When fiber was split into cereal-derived and fruit and vegetable-derived, cereal-derived fiber decreased while fruit and vegetable-derived fiber increased in both genders (Table 2 and Table S2). 

**Table 2:** Total energy and nutrient intakes of the participants of the “Bus Santé” study, Geneva, Switzerland, according to study period, stratified by gender.

			Men							Women				
	1993–1999		2000–2005		2006–2012			1993–1999		2000–2005		2006–2012		
Sample Size	3257		2859		2138			3665		3175		2169		
	Mean	SE	Mean	SE	Mean	SE	*p*-Value	Mean	SE	Mean	SE	Mean	SE	*p*-Value
Total energy (Kcal/day)	2195	11	2185	12	2104	14	<0.001	1808	9	1799	10	1734	12	<0.001
Macronutrients (g/day)														
Proteins														
Total	82.5	0.3	83.5	0.3	83.8	0.3	0.005	69.6	0.2	70.0	0.2	70.0	0.3	0.31
Vegetal	24.9	0.1	25.1	0.1	25.2	0.1	0.35	21.6	0.1	21.5	0.1	21.8	0.1	0.04
Animal	57.6	0.3	58.4	0.3	58.7	0.4	0.06	48.0	0.3	48.6	0.3	48.2	0.3	0.33
Carbohydrates														
Total	239	1	241	1	244	1	0.002	205	1	205	1	208	1	0.02
Mono/disaccharides	102	1	104	1	108	1	<0.001	99	0.6	101	1	103	1	0.003
Polysaccharides	136	1	136	1	135	1	0.66	106	1	104	1	105	1	0.12
Fibers														
Total	16.1	0.1	16.3	0.1	16.6	0.1	<0.001	16.6	0.1	16.5	0.1	16.8	0.1	0.34
Cereal	8.6	0.1	8.7	0.1	8.3	0.1	0.03	7.8	0.1	7.3	0.1	7.3	0.1	<0.001
Fruits and vegetables	6.8	0.1	7.1	0.1	7.7	0.1	<0.001	8.3	0.1	8.7	0.1	9.0	0.1	<0.001
Fat														
Total	82.7	0.3	82.5	0.3	83.2	0.4	0.36	70.2	0.2	70.6	0.3	70.4	0.3	0.48
SFA	32.2	0.1	31.6	0.2	31.2	0.2	<0.001	25.6	0.1	25.3	0.1	24.8	0.1	<0.001
MUFA	31.5	0.1	32.3	0.2	33.4	0.2	<0.001	27.6	0.1	28.8	0.1	29.3	0.2	<0.001
PUFA	12.5	0.1	12	0.1	11.8	0.1	<0.001	10.9	0.1	10.4	0.1	10.0	0.1	<0.001
Micronutrients														
Cholesterol (mg/day)	358	2	357	2	361	3	0.60	294	2	290	2	298	2	<0.05
Calcium (mg/day)	1178	8	1188	9	1126	10	<0.001	1058	6	1058	7	1008	8	<0.001
Iron (mg/day)	12.2	0.04	12.1	0.04	12.1	0.04	0.13	10.4	0.03	10.3	0.03	10.2	0.04	<0.001
Vitamin A (μg RAE/day)	924	10	869	11	887	12	<0.001	893	9	856	10	848	12	0.004
Retinol (µg/day)	651	9	580	10	570	11	<0.001	560	8	501	9	455	11	<0.001
Carotene (µg/day)	3283	44	3465	47	3812	55	<0.001	3990	54	4262	58	4714	71	<0.001
Vitamin D (µg/day)	2.86	0.03	2.83	0.04	2.95	0.04	0.09	2.76	0.03	2.67	0.03	2.84	0.04	0.003
Alcohol (g/day) *	21	0.4	19.6	0.4	16.8	0.4	<0.001	9.2	0.2	8.4	0.2	7.1	0.3	<0.001

Results are expressed as multivariable-adjusted means and standard error (SE). RAE, retinol activity equivalent. SFA, saturated fatty acids; MUFA, monounsaturated fatty acids; PUFA, polyunsaturated fatty acids. Comparisons between periods were performed by ANOVA adjusting for age, marital status, country of birth (dichotomized into Switzerland yes/no), smoking and total energy intake (for nutrients). *, all subjects.

**Figure 1:**
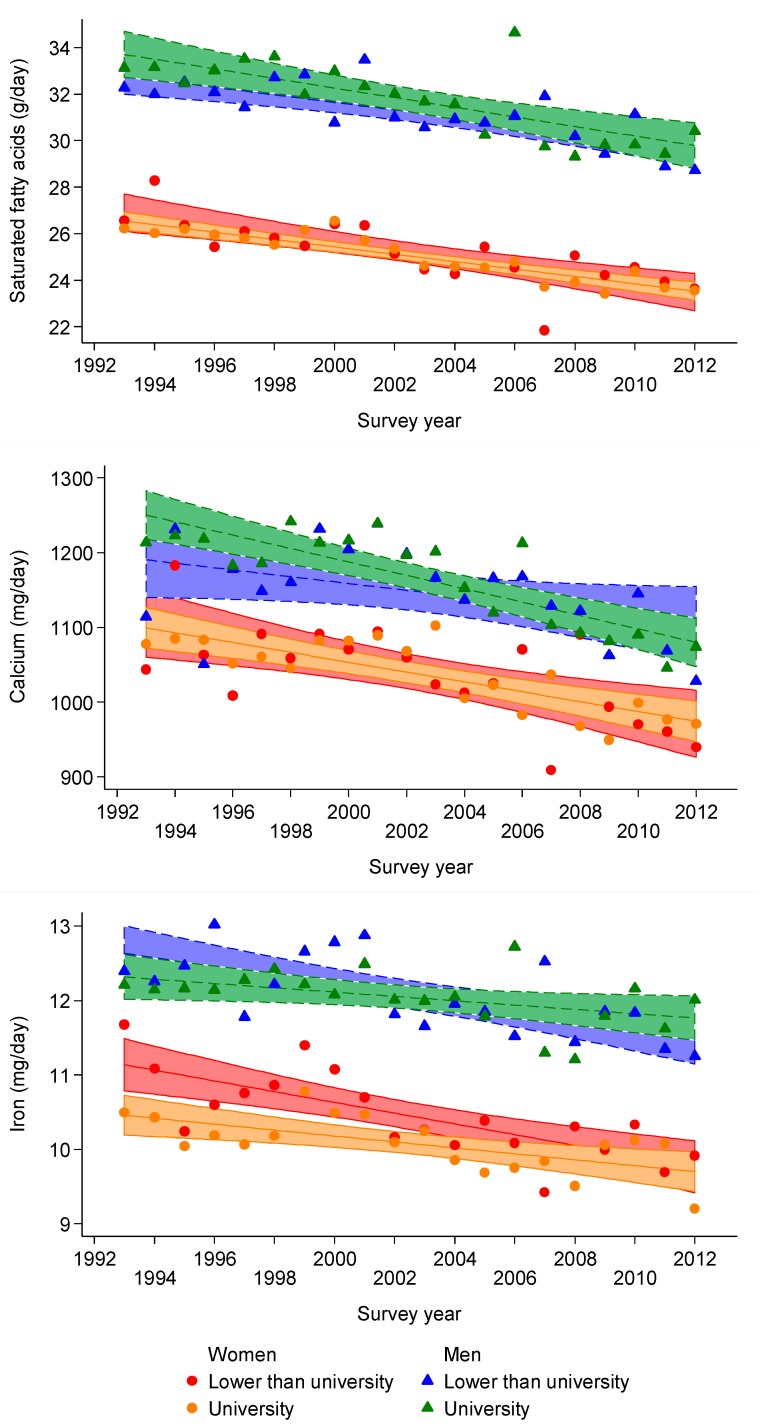
Unadjusted trends for saturated fatty acids (**upper panel**), calcium (**middle panel**) and iron (**lower panel**) according to gender and educational level, 1993–2012, Geneva, Switzerland.

Trends in TEI and nutrient intake overall and according to high or low educational level in men are summarized in Table S2a (unadjusted or energy-adjusted for nutrients) and in Table 3 (a) (multivariable adjusted). Total energy, SFA, poly-unsaturated fatty acids (PUFA), calcium, retinol and alcohol intake decreased, while total carbohydrates, mono/disaccharide, MUFA and carotene increased in all groups. When fiber was split into cereal-derived and fruit and vegetable-derived fiber, opposite trends were found: cereal-derived fiber decreased while fruit and vegetable-derived fiber increased. No significant interactions with education were found for TEI and most nutrients, except a trend towards stronger decreases in SFA and calcium intake among participants with non-university level (Figure 1 and Table 3 (a)).

Trends in TEI and nutrient intake overall and according to high or low educational level in women are summarized in Table S3b (unadjusted or energy-adjusted for nutrients) and in Table 3 (b) (multivariable adjusted). Total energy, SFA, PUFA, calcium, iron, vitamin A, retinol and alcohol intake decreased, while mono/disaccharide, MUFA and carotene increased. When fiber was split into cereal-derived and fruit and vegetable-derived fiber, opposite trends were found: cereal-derived fiber decreased, while fruit and vegetable-derived fiber increased. The decrease in iron intake tended to be stronger among women with a university level (*p* for interaction = 0.002) (Figure 1 and Table 3 (b)).

**Table 3:** Trends in total energy and nutrient intakes in the “Bus Santé” study, Geneva, Switzerland, 1993–2012, overall and according to high or low educational level

	All	*p*-Value		High		*p*-Value		Low		*p*-Value		*p*-Value §		*p*-Value §§	
(a): Men															
Sample Size	8254			3006				5248							
Total energy (Kcal/day/year)	−6.9 (−9.4; −4.3)	<0.001		−9.6 (−13.6; −5.6)		<0.001		−4.6 (−8.0; −1.3)		0.006		0.06		0.053	
Macronutrients (g/day/year)															
Proteins															
Total	0.12 (0.06; 0.18)	<0.001		0.16 (0.06; 0.25)		0.001		0.09 (0.01; 0.18)		0.03		0.43		0.40	
Vegetal	0.02 (−0.01; 0.04)	0.14		0.00 (−0.04; 0.04)		0.98		0.02 (−0.01; 0.06)		0.18		0.17		0.29	
Animal	0.10 (0.03; 0.17)	0.004		0.16 (0.05; 0.27)		0.005		0.07 (−0.02; 0.16)		0.13		0.23		0.26	
Carbohydrates	0.34 (0.15; 0.53)	<0.001		0.21 (−0.08; 0.50)		0.16		0.44 (0.19; 0.69)		<0.001		0.11		0.21	
Mono/disaccharides	0.40 (0.25; 0.55)	<0.001		0.35 (0.11; 0.58)		0.004		0.46 (0.26; 0.66)		<0.001		0.50		0.56	
Polysaccharides	−0.05 (−0.22; 0.11)	0.52		−0.14 (−0.40; 0.12)		0.30		−0.01 (−0.23; 0.20)		0.92		0.20		0.35	
Fibers (g/day/year)															
Total	0.03 (0.01; 0.06)	0.02		0.01 (−0.04; 0.05)		0.79		0.04 (0.00; 0.07)		0.03		0.12		0.22	
Cereal	−0.03 (−0.05; −0.01)	0.01		−0.05 (−0.08; −0.01)		0.01		−0.02 (−0.05; 0.01)		0.12		0.13		0.18	
Fruits and vegetables	0.06 (0.05; 0.08)	<0.001		0.06 (0.03; 0.08)		<0.001		0.06 (0.04; 0.09)		<0.001		0.35		0.65	
Fat (g/day/year)															
Total	0.03 (−0.03; 0.10)	0.30		0.06 (−0.04; 0.16)		0.24		0.00 (−0.08; 0.09)		0.97		0.14		0.42	
SFA	−0.08 (−0.11; −0.04)	<0.001		−0.03 (−0.08; 0.02)		0.20		−0.11 (−0.15; −0.06)		<0.001		0.002		0.03	
MUFA	0.15 (0.11; 0.18)	<0.001		0.13 (0.08; 0.19)		<0.001		0.14 (0.10; 0.18)		<0.001		0.95		0.78	
PUFA	−0.06 (−0.07; −0.04)	<0.001		−0.07 (−0.09; −0.04)		<0.001		−0.05 (−0.07; −0.03)		<0.001		0.47		0.47	
Micronutrients															
Cholesterol (mg/day/year)	0.29 (−0.17; 0.75)	0.22		0.54 (−0.22; 1.30)		0.16		0.06 (−0.53; 0.64)		0.85		0.36		0.39	
Calcium (mg/day/year)	−3.45 (−5.34; −1.57)	<0.001		−1.03 (−3.84; 1.78)		0.47		−5.24 (−7.78; −2.70)		<0.001		0.005		0.03	
Iron (mg/day/year)	−0.01 (−0.02; 0.00)	0.08		−0.02 (−0.03; −0.001)		0.01		−0.01 (−0.02; 0.01)		0.35		0.10		0.15	
Vitamin A (μg RAE/day)	−3.45 (−5.66; −1.23)	0.002		−3.57 (−6.91; −0.23)		0.04		−3.67 (−6.63; −0.70)		0.02		0.82		0.90	
Retinol (µg/day/year)	−6.57 (−8.63; −4.50)	<0.001		−6.24 (−9.34; −3.14)		<0.001		−6.74 (−9.52; −3.96)		<0.001		0.65		0.75	
Carotene (µg/day/year)	37.4 (27.6; 47.3)	<0.001		32.1 (15.5; 48.6)		<0.001		36.9 (24.5; 49.2)		<0.001		0.59		0.65	
Vitamin D (µg/day/year)	0.01 (0.00; 0.01)	0.08		0.01 (−0.01; 0.02)		0.33		0.00 (−0.01; 0.01)		0.85		0.53		0.52	
Alcohol (g/day/year)	−0.31 (−0.39; −0.24)	<0.001		−0.29 (−0.39; −0.18)		<0.001		−0.32 (−0.43; −0.21)		<0.001		0.70		0.56	
**(b): Women**															
**Sample Size**	**9009**				**3082**				**5927**						
Total energy (Kcal/day/year)	−5.3 (−7.4; −3.1)		<0.001		−7.5 (−11.1; −3.8)		<0.001		−4.6 (−7.3; −1.9)		0.001		0.20		0.21
Macronutrients (g/day/year)															
Proteins															
Total	0.03 (−0.02; 0.09)		0.20		−0.04 (−0.13; 0.05)		0.39		0.07 (0.01; 0.14)		0.03		0.06		0.06
Vegetal	0.02 (−0.005; 0.04)		0.13		0.02 (−0.01; 0.06)		0.19		0.01 (−0.02; 0.04)		0.56		0.50		0.56
Animal	0.02 (−0.04; 0.08)		0.54		−0.06 (−0.16; 0.04)		0.23		0.07 (−0.01; 0.15)		0.09		0.06		0.07
Carbohydrates	0.18 (0.03; 0.34)		0.02		0.28 (0.03; 0.54)		0.03		0.16 (−0.04; 0.35)		0.12		0.39		0.51
Mono/disaccharides	0.27 (0.13; 0.41)		<0.001		0.23 (0.00; 0.46)		0.05		0.31 (0.13; 0.49)		0.001		0.70		0.57
Polysaccharides	−0.08 (−0.22; 0.06)		0.27		0.06 (−0.17; 0.29)		0.62		−0.15 (−0.32; 0.03)		0.10		0.18		0.19
Fibers (g/day/year)															
Total	0.01 (−0.02; 0.03)		0.54		0.00 (−0.04; 0.04)		0.90		0.01 (−0.02; 0.04)		0.65		0.68		0.63
Cereal	−0.04 (−0.06; −0.03)		<0.001		−0.05 (−0.08; −0.02)		<0.001		−0.05 (−0.07; −0.02)		<0.001		0.92		0.86
Fruits and vegetables	0.06 (0.04; 0.08)		<0.001		0.05 (0.02; 0.08)		<0.001		0.06 (0.03; 0.08)		<0.001		0.58		0.61
Fat (g/day/year)															
Total	0.01 (−0.04; 0.07)		0.68		0.04 (−0.05; 0.14)		0.39		0.00 (−0.07; 0.07)		0.94		0.39		0.44
SFA	−0.07 (−0.09; −0.04)		<0.001		−0.05 (−0.09; −0.01)		0.03		−0.07 (−0.11; −0.04)		<0.001		0.22		0.36
MUFA	0.14 (0.11; 0.17)		<0.001		0.15 (0.10; 0.20)		<0.001		0.13 (0.09; 0.17)		<0.001		0.71		0.68
PUFA	−0.08 (−0.09; −0.06)		<0.001		−0.07 (−0.09; −0.05)		<0.001		−0.07 (−0.09; −0.06)		<0.001		0.88		0.76
Micronutrients															
Cholesterol (mg/day/year)	0.25 (−0.16; 0.66)		0.23		0.36 (−0.37; 1.10)		0.33		0.17 (−0.32; 0.67)		0.49		0.77		0.69
Calcium (mg/day/year)	−3.69 (−5.19; −2.19)		<0.001		−3.83 (−6.27; −1.38)		0.002		−3.68 (−5.59; −1.77)		<0.001		0.73		0.97
Iron (mg/day/year)	−0.02 (−0.02; −0.01)		<0.001		−0.03 (−0.04; −0.02)		<0.001		−0.01 (−0.02; 0.00)		0.04		0.001		0.002
Vitamin A (μg RAE/day)	−4.47 (−6.62; −2.31)		<0.001		−4.24 (−7.73; −0.76)		0.02		−4.54 (−7.30; −1.78)		0.02		0.87		0.85
Retinol (µg/day/year)	−8.93 (−10.9; −7.01)		<0.001		−8.57 (−11.5; −5.61)		<0.001		−8.76 (−11.3; −6.25)		<0.001		0.81		0.78
Carotene (µg/day/year)	53.6 (41.0; 66.2)		<0.001		51.9 (28.9; 74.9)		<0.001		50.7 (35.7; 65.7)		<0.001		0.92		0.90
Vitamin D (µg/day/year)	0.01 (0.00; 0.01)		0.13		0.01 (−0.01; 0.02)		0.40		0.00 (−0.01; 0.01)		0.58		0.90		0.82
Alcohol (g/day/year)	−0.14 (−0.18; −0.09)		<0.001		−0.19 (−0.27; −0.12)		<0.001		−0.13 (−0.18; −0.07)		<0.001		0.10		0.23

SFA, saturated fatty acids; MUFA, monounsaturated fatty acids; PUFA, poly-unsaturated fatty acids; RAE, retinol activity equivalent. Results are expressed as multivariable-adjusted yearly change and (95% confidence interval). § *p*-value for the interaction between survey year and education, unadjusted or adjusting for total energy intake (for nutrients); §§ *p*-value for the interaction between survey year and education, adjusting for age, marital status, country of birth (dichotomized into Switzerland yes/no), smoking and total energy intake (for nutrients). Statistical analyses by linear regression using yearly data adjusting for age, marital status, country of birth, smoking and total energy intake (for nutrients).

### 3.3. Educational Differences in Nutrient Intake

Differences between educational level (low *versus* high) regarding nutrient intake for study periods 1993–1999 and 2006–2012 are summarized in Table S4 (age-adjusted, and also energy-adjusted for nutrients) and Table 4 (multivariable adjusted). 

**Table 4:** Multivariable-adjusted differences in total energy and nutrient intake between educational levels in the “Bus Santé” study, Geneva, Switzerland, 1993–1999 and 2006–2012, according to gender and survey period.

	Men				Women			
	1993–1999	*p*-Value	2006–2012	*p*-Value	1993–1999	*p*-Value	2006–2012	*p*-Value
Sample Size	3257		2138		3665		2169	
Total energy (Kcal/day)	24.3 (−24.7; 73.3)	0.33	70.6 (13.8; 127.4)	0.02	−55.8 (−97.3; −14.3)	0.008	−25.6 (−75.4; 24.1)	0.31
Macronutrients (g/day)								
Proteins								
Total	−0.20 (−1.35; 0.96)	0.74	−1.06 (−2.52; 0.39)	0.15	−0.12 (−1.12; 0.88)	0.82	0.79 (−0.53; 2.12)	0.24
Vegetal	−0.45 (−0.93; 0.03)	0.07	−0.26 (−0.82; 0.29)	0.36	−0.42 (−0.83; −0.01)	0.04	−0.44 (−0.93; 0.06)	0.08
Animal	0.26 (−1.06; 1.57)	0.70	−0.80 (−2.46; 0.86)	0.34	0.31 (−0.84; 1.45)	0.60	1.23 (−0.28; 2.74)	0.11
Carbohydrates								
Total	0.78 (−2.81; 4.36)	0.67	1.56 (−2.47; 5.59)	0.45	1.94 (−1.03; 4.91)	0.20	2.87 (−0.55; 6.30)	0.10
Mono/disaccharides	0.50 (−2.41; 3.41)	0.74	−0.20 (−3.56; 3.16)	0.91	0.05 (−2.6; 2.71)	0.97	2.22 (−1.02; 5.47)	0.18
Polysaccharides	0.12 (−3.05; 3.29)	0.94	1.71 (−1.88; 5.29)	0.35	1.80 (−0.88; 4.49)	0.19	0.57 (−2.56; 3.71)	0.72
Fiber								
Total	−0.77 (−1.27; −0.27)	0.002	−0.69 (−1.27; −0.10)	0.02	−0.82 (−1.28; −0.36)	<0.001	−0.52 (−1.08; 0.04)	0.07
Cereal	−0.60 (−1.01; −0.20)	0.004	−0.37 (−0.82; 0.08)	0.10	−0.52 (−0.87; −0.18)	<0.001	−0.35 (−0.75; 0.05)	0.08
Fruits and vegetables	−0.12 (−0.43; 0.20)	0.47	−0.22 (−0.63; 0.20)	0.31	−0.28 (−0.63; 0.06)	0.11	−0.13 (−0.58; 0.31)	0.55
Fat								
Total	−1.66 (−2.92; −0.40)	0.01	−1.76 (−3.15; −0.37)	0.01	0.48 (−0.58; 1.53)	0.38	−1.14 (−2.37; 0.08)	0.07
SFA	−0.19 (−0.82; 0.45)	0.56	−0.92 (−1.59; −0.25)	0.007	0.48 (−0.04; 0.99)	0.07	−0.28 (−0.84; 0.29)	0.34
MUFA	−1.17 (−1.77; −0.57)	<0.001	−0.88 (−1.61; −0.16)	0.02	−0.21 (−0.76; 0.33)	0.45	−1.02 (−1.72; −0.31)	0.005
PUFA	−0.08 (−0.42; 0.25)	0.63	0.29 (−0.04; 0.62)	0.09	0.28 (0.01; 0.54)	0.04	0.25 (−0.01; 0.52)	0.06
Micronutrients								
Cholesterol (mg/day)	−7.71 (−16.3; 0.89)	0.08	−9.22 (−19.6; 1.10)	0.08	−0.03 (−7.73; 7.67)	0.99	−5.5 (−16.14; 5.14)	0.31
Calcium (mg/day)	22.9 (−14.7; 60.5)	0.23	−46.9 (−85.5; −8.2)	0.02	0.96 (−28.4; 30.3)	0.95	−15.0 (−47.6; 17.6)	0.37
Iron (mg/day)	−0.29 (−0.45; −0.13)	<0.003	−0.13 (−0.30; 0.05)	0.15	−0.38 (−0.51; −0.24)	<0.001	−0.06 (−0.21; 0.10)	0.48
Vitamin A (μg RAE/day)	−27.6 (−70.8; 15.6)	0.21	−9.9 (−58.7; 38.9)	0.69	−11.5 (−54.5; 31.6)	0.60	−37.8 (−82.1; 6.6)	0.10
Retinol (µg/day)	−2.4 (−43.2; 38.5)	0.91	10.0 (−35.4; 55.5)	0.67	22.3 (−17.0; 61.6)	0.27	−8.7 (−46.6; 29.2)	0.65
Carotene (µg/day)	−303 (−480; −125)	<0.001	−239 (−470; −8)	0.04	−406 (−628; −183)	<0.001	−349 (−667; −31)	0.03
Vitamin D (µg/day)	−0.46 (−0.60; −0.31)	<0.001	−0.52 (−0.70; −0.35)	<0.001	−0.30 (−0.43; −0.17)	<0.001	−0.30 (−0.47; −0.13)	<0.001
Alcohol	1.87 (0.31; 3.43)	0.02	1.92 (0.40; 3.44)	0.01	−1.64 (−2.59; −0.69)	0.001	−0.65 (−1.51; 0.20)	0.13

SFA, saturated fatty acids; MUFA, monounsaturated fatty acids; PUFA, poly-unsaturated fatty acids; RAE, retinol activity equivalent. Results are expressed as multivariable adjusted differences (low *vs.* high) and (95% confidence interval) in nutrient intake between low and high educational categories. Statistical analysis by analysis of variance adjusting for age, country of birth (dichotomized into Switzerland yes/no), marital status, smoking status and total energy intake (for nutrients).

Compared to men with a low education, men with a high education had higher MUFA, carotene and vitamin D intakes in 1993–1999, but the differences for MUFA and carotene became non-significant in 2006–2012. A trend for a higher fiber (total, cereal or fruit and vegetable derived) and iron intake in men with a high education was also found in 1993–1999, and a trend for a higher SFA intake in 2006–2012 (Table 4).

Compared to women with a low education, women with a high education had higher fiber (total, cereal or fruit and vegetable derived), iron, carotene, vitamin D and alcohol intakes in 1993–1999, but these differences (vitamin D excepted) were no longer significant in 2006–2012. A trend for a higher MUFA intake in women with a high education was also found in 2006–2012 (Table 4).

### 3.4. Sensitivity Analyses

The results regarding the trends as well as the educational differences in total energy and nutrient intake remained after including participants with extreme energy intakes (Tables S5 and S6).

## 4. Discussion

To our knowledge, this is the longest study ever conducted in Switzerland and one of the few in Europe assessing trends in dietary intakes according to educational level in a representative, population-based sample. Our results indicate that dietary intake evolved similarly in both educational groups, with a trend towards a sharper decrease in SFA and calcium intake among men with a low education and a sharper decrease in iron intake among women with a high education. Our results also suggest that educational differences in dietary intake tend to decrease with time, but that several educational differences were still observed at the end of the study period, especially among men.

### 4.1. Trends in Energy and Nutrient Intake

Total energy intake decreased in both genders and educational groups, a finding also reported previously [14,20]. Still, this decrease was rather small (90 kcal/day for the whole study period) and might have no significant impact in the general population. Further, a slight but significant increase in body mass index (annual increase of 0.04 kg/m^2^ for men and for women) was found. Possible reasons include a decrease in energy expenditure and an increase in underreporting. Indeed, the proportion of under-reporters increased from 2.6% in 1993–1999 to 3.5% in 2006–2012 (*p* < 0.001), but no difference was found between under-reporters and included participants regarding body mass index (Table S7). Still, as energy intakes below 850 kcal/day were excluded from the analysis, the effect of underreporting might be small. Another likely reason is that the same FFQ was used for 20 years; hence, it is likely that many processed foods that have been available only recently were missed. Overall, our results indicate that the decrease in total energy intake in the Geneva population between 1993 and 2012 is small and would have little if no effect on health.

Mono/disaccharide intake increased in men only, and this increase was similar for both educational groups. This finding is in agreement with other studies [20] and with the increase in sugar availability observed in Switzerland for the period 1961–2007 [10]. The fact that this increase was found only in men might be related to their stronger preference for sweet fat food [24]. Although our findings are in agreement with other studies using data from the FAO, it would be of interest that they be replicated in other Swiss cantons using different methodologies.

Although no changes were observed in total fat intake, significant changes were found in the types of fatty acid intake. SFA intake decreased in both educational groups among women and in the low educated group among men; MUFA intake increased and PUFA intake decreased in both genders and both educational groups, a finding already noted previously [20]. Possible explanations include an increased awareness of the harmful effects of SFA to cardiovascular health and a change in fat availability in Switzerland; indeed, sunflower oil and olive oil availability increased while palm oil decreased in Switzerland in the last decades [10]. Conversely, the decrease in PUFA is more difficult to explain: although the annual per capita fish supply increased [25,26], still the estimated corresponding fat supply quantity remained stable at 1.2 g/day [25]. A possible explanation is the fact that 95% of the fish consumed in Switzerland is imported, with a significant portion of farmed fish, whose fatty acid composition is less favorable than wild fish [27], although this statement has been challenged [28]. Another possibility is the increasing consumption of farmed pangasius fish [29], whose eicosapentaenoic and docosahexaenoic acid content is considerably lower than that of other fish [30]. Overall, our results indicate that fatty acid consumption changed significantly in Switzerland among both genders and educational levels, and that these changes might be related to changes in food availability.

Calcium intake decreased in both genders. These findings are in agreement with a previous study [20], and a possible explanation would be a lower consumption of dairy products [10], which could also partly account for the decrease in SFA. Indeed, the amount of calcium from dairy products decreased from 655 mg/day in 1993–1999 to 593 mg/day in 2006–2012 among men and from 536 mg/day to 496 mg/day among women (both *p* < 0.001) with a corresponding decrease in the percentage of total calcium from dairy products (multivariate adjusted values: from 50% in 1993–1999 to 47% in 2006–2012 among men and from 47% to 45% among women, both *p* < 0.001). Interestingly, the decrease tended to be more pronounced among men with a low education, while in women the decreases were similar irrespective of the educational level. Although the reasons for a stronger decrease in men with a low education are currently unknown, our results suggest that promotion of calcium rich foods should be implemented in Switzerland, especially in men with a low education, in order to curb the decreasing trend and to prevent osteoporosis.

Total fiber intake showed few changes during the study period. Conversely, when total fiber was split into cereal-derived and fruit and vegetable-derived, opposite and significant trends were found for both genders and for both educational groups. Interestingly, the decrease in cereal-derived fiber consumption was compensated by an increase in fruit and vegetable fiber consumption. These findings are in agreement with another study using FAO data [10], which showed a decrease in cereal consumption and a mixed trend regarding fruit and vegetable consumption, with a decrease in fruit but an increase in vegetable consumption. As the impact of cereal-derived and fruit and vegetable-derived fiber differ slightly [31,32], the health effects of such changes should be further explored.

Iron intake decreased in women only, and this decrease was steeper among women with a high education. Interestingly, during the study period, iron from animal sources remained stable (from 2.1 mg/day in 1993–1999 to 2.1 mg/day in 2006–2012, *p* for trend = 0.25) while iron from vegetal sources decreased from 5.0 mg/day in 1993–1999 to 4.7 mg/day in 2006–2012, *p* for trend <0.001). This decreasing trend is also of concern, as the percentage of women with an adequate iron intake was only 8.4% in 2009 [33]. Thus, adequate dietary iron intake should be promoted in the Geneva population, namely among women.

Total vitamin A and retinol intake decreased while carotene intake increased in both genders; no specific effect of education was found. These findings are in agreement with previous studies which showed a decrease in beef and liver consumption [34] and an increased consumption of vegetables [10]. Overall, our results suggest that the decreased consumption of animal-derived retinol was not compensated by the increase in carotene intake from plant sources.

Alcohol intake decreased in both genders and in both educational groups, a finding in agreement with previous studies [10,20] and with sales of alcoholic beverages (Swiss Alcohol Board 2012) in Switzerland. Possible explanations include the increasing cost of alcoholic beverages and increasingly tighter legislation regarding alcohol consumption, namely regarding drivers [35,36].

### 4.2. Educational Differences in Nutrient Intake

In 1993–1999, participants with a low education had lower intakes of MUFA, fiber, iron, carotene and vitamin D than participants with a high education, a finding in agreement with the literature [37,38]. Although most of the differences became non-significant (at *p* < 0.01) in 2006–2012, still participants with a high education had a better dietary intake. A likely explanation is that lower educated groups consume more energy-dense and nutrient-poor diets [5], and that this behavior persisted throughout the study.

In 1993–1999, men with a high education had a lower alcohol intake, the opposite being found for women. Our results are in agreement with other studies [39,40], where women with a higher educational attainment were more often drinkers and more likely to drink heavily. As suggested elsewhere, such behavior might be considered as a symbol of greater gender equality, something that might be valued by higher educated groups [40,41]. Interestingly, the educational differences observed in 1993–1999 in women were no longer significant in 2006–2012, probably due to the decrease in alcohol consumption among women with a high education.

Overall, our results indicate that, despite a favorable trend regarding dietary intake, educational differences regarding the intake of several macro and micronutrient persist. Still, the observed differences were small and probably cannot explain the major inequalities observed in cardiovascular risk factors [42] and in morbidity and mortality [12] observed in Switzerland. However, the extent to which social differences in diet explain social inequalities in health should be explored in further studies.

### 4.3. Strengths and Limitations

Strengths of the study are the long study period (20 years), its low attrition rate and its representative sampling and design [43].

This study also has some limitations. Firstly, data were collected in one French-speaking Swiss canton, and the results might not be applicable to other German- or Italian-speaking cantons. Still, in the absence of objective data for other Swiss cantons, our results could serve as reference for future studies. Secondly, dietary intake was collected using a FFQ, which, as indicated previously might not capture all foods consumed, especially novel processed and non-staple foods preferred by younger generations and certain population groups. However, similar use of FFQ data has provided very important information on sodium [44,45], dietary fats and oils [17] consumption. Thirdly, the FFQ assesses the dietary of the previous 4 weeks and does not encompass a whole year. Still, we analyzed yearly data and the distribution of responders throughout the year did not change during the study period; hence, any issues related to seasonality would be cancelled. In addition, to our knowledge, there is no validated FFQ assessing annual dietary intake in Switzerland, and it has been shown that FFQs assessing dietary intake for shorter periods than one year have the same validity as FFQs assessing annual dietary intake [18]. Notwithstanding, some of the issues might have been addressed by the use of repeated 24 h recalls in addition to the FFQ. Fifthly, educational level was used as a proxy for SES, which could underestimate the burden of SES disparities on nutrition [13]. Indeed, other important SES determinants of dietary intake such as income were not available for the whole study period and could not be used. Still, education a frequently used SES indicator [46] and it is one of the socioeconomic indicators especially likely to capture aspects of lifestyle and behavior [47]. Sixthly, country of birth was dichotomized into Switzerland (yes/no), and this might not capture all the dietary variety of the over 30 different countries of birth included in our sample. Still, splitting the countries would lead to small sample sizes that might preclude adjustment, and further studies should be conducted on trends in dietary intake among migrants in Switzerland. Finally, the same questionnaire was used throughout the study, which might lead to a decrease in its ability to adequately capture dietary intake. Still, to our knowledge, there is no gold standard to assess long-term trends in dietary intake, and using different FFQs wouldn’t allow comparisons over time.

## 5. Conclusions

We conclude that, in Switzerland, dietary intake evolved similarly between 1993 and 2012 in both genders and educational groups. Although most educational differences observed in 1993–1999 were no longer significant in 2006–2012, participants with a high education still tended to have a better dietary intake (higher MUFA, fiber, iron, carotene and vitamin D intake) than participants with a low education.

## Supplementary Materials

Supplementary materials can be accessed at: http://www.mdpi.com/2072-6643/7/11/5481/s1.
