# Supplementary material for: Dietary Intake according to Gender and Education: A Twenty-Year Trend in a Swiss Adult Population
Source: Nutrients. 2015 Nov 18;7(11):9558–72. doi: 10.3390/nu7115481 (PMC4663609; doi:10.3390/nu7115481)
Supplement: Supplementary file 1 [file nutrients-07-05481-s001.docx]

**Supplementary Materials: Dietary Intake according to Gender and Education: A Twenty-Year Trend in a Swiss Adult Population**

Pedro Marques-Vidal ^1,^*, Eirini Rousi ^2^, Fred Paccaud ^2^, Jean-Michel Gaspoz ^3^, Jean-Marc Theler ^3^, Murielle Bochud ^2^, Silvia Stringhini ^2^ and Idris Guessous ^2,3,4^

**Figure S1.** Selection procedure.

**Table S1.** Comparison between excluded and included participants.

|  | **Included** | | **Excluded** | | ***p*-Value** |
| --- | --- | --- | --- | --- | --- |
| **N** | **17,263** | | **1880** | |  |
| Age (years) | 52.0 | (10.6) | 47.9 | (13.8) | <0.001 |
| BMI (kg/m^2^) | 24.7 | (4.0) | 24.6 | (4.2) | 0.10 |
| Gender |  |  |  |  |  |
| Male | 8254 | (47.8) | 831 | (44.1) | 0.002 |
| Female | 9009 | (52.2) | 1053 | (55.9) |  |
| Education |  |  |  |  |  |
| High | 6088 | (35.3) | 561 | (34.8) | 0.70 |
| Low | 11,175 | (64.7) | 1052 | (65.2) |  |
| Country of birth |  |  |  |  |  |
| Switzerland | 9498 | (55.0) | 914 | (48.5) | <0.001 |
| Other | 7765 | (45.0) | 970 | (51.5) |  |
| Marital status |  |  |  |  |  |
| Single | 1684 | (9.8) | 334 | (18.0) |  |
| Married/cohabitating | 12,675 | (73.4) | 1187 | (64.0) | <0.001 |
| Divorced | 2283 | (13.2) | 253 | (13.7) |  |
| Widowed | 621 | (3.6) | 80 | (4.3) |  |
| Smoking status |  |  |  |  |  |
| Never | 7775 | (45.0) | 869 | (47.7) |  |
| Former | 5538 | (32.1) | 447 | (24.5) | <0.001 |
| Current | 3950 | (22.9) | 507 | (27.8) |  |

Results are expressed as number of participants (%) or as mean (standard deviation). BMI, body mass index. Statistical comparisons by chi-square or student’s *t*-test.

**Table S2.** Total energy and dietary intakes of the participants of the “Bus Santé” study, Geneva, Switzerland, according to study period, stratified by gender.

|  |  | | **Men** | |  | |  |  | | **Women** | |  | |  |
| --- | --- | --- | --- | --- | --- | --- | --- | --- | --- | --- | --- | --- | --- | --- |
|  | **1993–1999** | | **2000–2005** | | **2006–2012** | | ***p*-Value** | **1993–1999** | | **2000–2005** | | **2006–2012** | | ***p*-Value** |
| **Sample Size** | **3257** | | **2859** | | **2138** | |  | **3665** | | **3175** | | **2169** | |  |
|  | Mean | SE | Mean | SE | Mean | SE |  | Mean | SE | Mean | SE | Mean | SE |  |
| Total energy (Kcal/day) | 2197 | 649 | 2184 | 645 | 2103 | 662 | <0.001 | 1809 | 574 | 1800 | 564 | 1733 | 567 | <0.001 |
| Macronutrients (g/day) |  |  |  |  |  |  |  |  |  |  |  |  |  |  |
| Proteins, total | 82.5 | 0.3 | 83.5 | 0.3 | 83.9 | 0.3 | <0.001 | 69.5 | 0.2 | 70.0 | 0.2 | 70.0 | 0.3 | 0.26 |
| Vegetal | 24.9 | 0.1 | 25.1 | 0.1 | 25.3 | 0.1 | 0.08 | 21.5 | 0.1 | 21.5 | 0.1 | 21.9 | 0.1 | 0.02 |
| Animal | 57.6 | 0.3 | 58.4 | 0.3 | 58.6 | 0.4 | 0.09 | 48.0 | 0.3 | 48.6 | 0.3 | 48.1 | 0.3 | 0.33 |
| Carbohydrates, total | 238 | 1 | 241 | 1 | 245 | 1 | <0.001 | 205 | 1 | 205 | 1 | 208 | 1 | 0.02 |
| Mono/disaccharides | 102 | 1 | 104 | 1 | 108 | 1 | <0.001 | 99 | 1 | 101 | 1 | 103 | 1 | 0.002 |
| Polysaccharides | 136 | 1 | 136 | 1 | 136 | 1 | 0.94 | 106 | 1 | 104 | 1 | 105 | 1 | 0.14 |
| Fibres |  |  |  |  |  |  |  |  |  |  |  |  |  |  |
| Total | 16.0 | 0.1 | 16.3 | 0.1 | 16.7 | 0.2 | <0.05 | 16.5 | 0.1 | 16.5 | 0.1 | 16.9 | 0.1 | 0.09 |
| Cereal | 8.6 | 0.1 | 8.7 | 0.1 | 8.4 | 0.1 | 0.06 | 7.8 | 0.1 | 7.3 | 0.1 | 7.3 | 0.1 | <0.001 |
| Fruits and vegetables | 6.8 | 0.1 | 7.1 | 0.1 | 7.8 | 0.1 | <0.001 | 8.2 | 0.1 | 8.8 | 0.1 | 9.1 | 0.1 | <0.001 |
| Total fat | 82.9 | 0.3 | 82.4 | 0.3 | 83.0 | 0.4 | 0.45 | 70.3 | 0.2 | 70.5 | 0.3 | 70.3 | 0.3 | 0.73 |
| SFA | 32.3 | 0.1 | 31.6 | 0.2 | 31.0 | 0.2 | <0.001 | 25.7 | 0.1 | 25.2 | 0.1 | 24.8 | 0.2 | <0.001 |
| MUFA | 31.5 | 0.1 | 32.3 | 0.2 | 33.4 | 0.2 | <0.001 | 27.6 | 0.1 | 28.8 | 0.1 | 29.3 | 0.2 | <0.001 |
| PUFA | 12.5 | 0.1 | 12.0 | 0.1 | 11.8 | 0.1 | <0.001 | 11.0 | 0.1 | 10.4 | 0.1 | 10.0 | 0.1 | <0.001 |
| Micronutrients |  |  |  |  |  |  |  |  |  |  |  |  |  |  |
| Cholesterol (mg/day) | 358 | 2 | 358 | 2 | 360 | 3 | 0.72 | 294 | 2 | 290 | 2 | 298 | 2 | <0.05 |
| Calcium (mg/day) | 1180 | 9 | 1187 | 9 | 1123 | 11 | <0.001 | 1058 | 7 | 1058 | 7 | 1009 | 9 | <0.001 |
| Iron (mg/day) | 12.1 | 0.04 | 12.1 | 0.04 | 12.1 | 0.04 | 0.19 | 10.4 | 0.03 | 10.3 | 0.03 | 10.2 | 0.04 | <0.001 |
| Vitamin A (μg RAE/day) | 924 | 10 | 869 | 11 | 887 | 12 | <0.001 | 892 | 9 | 856 | 10 | 850 | 12 | 0.007 |
| Retinol (µg/day) | 652 | 9 | 581 | 10 | 568 | 11 | <0.001 | 560 | 8 | 501 | 9 | 456 | 11 | <0.001 |
| Carotene (µg/day) | 3272 | 44 | 3458 | 47 | 3836 | 55 | <0.001 | 3981 | 55 | 4262 | 59 | 4728 | 71 | <0.001 |
| Vitamin D (µg/day) | 2.85 | 0.03 | 2.83 | 0.04 | 2.96 | 0.04 | 0.06 | 2.75 | 0.03 | 2.67 | 0.03 | 2.85 | 0.04 | 0.002 |
| Alcohol (g/day) * | 21.1 | 0.4 | 19.7 | 0.4 | 16.5 | 0.4 | <0.001 | 9.1 | 0.2 | 8.5 | 0.2 | 7.1 | 0.3 | <0.001 |

Results are expressed as unadjusted or total energy adjusted means (standard deviation) for total energy intake or (standard error) for nutrients. RAE, retinol activity equivalent. Between period comparisons by ANOVA adjusting for total energy intake (for nutrients). * All subjects.

**Table S3.** Trends in total energy and nutrient intakes in the “Bus Santé” study, Geneva, Switzerland, 1993–2012, overall and according to high or low educational level.

|  | **All** | ***p*-Value** | **High** | ***p*-Value** | **Low** | ***p*-Value** |
| --- | --- | --- | --- | --- | --- | --- |
| **(a): Men** | | | | | | |
| **Sample Size** | **8254** |  | **3006** |  | **5248** |  |
| Total energy (Kcal/day/year) | −7.0 (−9.5; −4.5) | <0.001 | −9.5 (−13.5; −5.5) | <0.001 | −4.6 (−7.9; −1.3) | 0.007 |
| Macronutrients (g/day/year) |  |  |  |  |  |  |
| Proteins, total | 0.13 (0.06; 0.19) | <0.001 | 0.15 (0.05; 0.25) | 0.002 | 0.09 (0.01; 0.18) | 0.02 |
| Vegetal | 0.03 (0.01; 0.06) | 0.02 | 0.00 (−0.04; 0.04) | 0.88 | 0.04 (0.00; 0.07) | 0.04 |
| Animal | 0.10 (0.03; 0.17) | 0.008 | 0.15 (0.04; 0.26) | 0.009 | 0.06 (−0.03; 0.15) | 0.23 |
| Carbohydrates | 0.45 (0.26; 0.64) | <0.001 | 0.24 (−0.06; 0.54) | 0.11 | 0.55 (0.30; 0.79) | <0.001 |
| Mono/disaccharides | 0.45 (0.30; 0.61) | <0.001 | 0.37 (0.13; 0.61) | 0.002 | 0.49 (0.28; 0.69) | <0.001 |
| Polysaccharides | 0.01 (−0.16; 0.17) | 0.92 | −0.13 (−0.39; 0.13) | 0.33 | 0.07 (−0.14; 0.29) | 0.50 |
| Fibres (g/day/year) |  |  |  |  |  |  |
| Total | 0.05 (0.02; 0.08) | <0.001 | 0.01 (−0.03; 0.06) | 0.58 | 0.05 (0.02; 0.09) | 0.003 |
| Cereal | −0.02 (−0.04; 0.001) | 0.052 | −0.05 (−0.08; −0.01) | 0.01 | −0.02 (−0.04; 0.01) | 0.21 |
| Fruits and vegetables | 0.07 (0.05; 0.09) | <0.001 | 0.06 (0.03; 0.09) | <0.001 | 0.08 (0.05; 0.10) | <0.001 |
| Total fat (g/day/year) | 0.01 (−0.06; 0.08) | 0.77 | 0.06 (−0.04; 0.16) | 0.22 | −0.05 (−0.13; 0.04) | 0.30 |
| SFA | −0.09 (−0.13; −0.06) | <0.001 | −0.03 (−0.08; 0.02) | 0.28 | −0.14 (−0.18; −0.10) | <0.001 |
| MUFA | 0.14 (0.11; 0.17) | <0.001 | 0.13 (0.08; 0.19) | <0.001 | 0.13 (0.09; 0.17) | <0.001 |
| PUFA | −0.06 (−0.07; −0.04) | <0.001 | −0.07 (−0.09; −0.04) | <0.001 | −0.05 (−0.08; −0.03) | <0.001 |
| Micronutrients |  |  |  |  |  |  |
| Cholesterol (mg/day/year) | 0.25 (−0.21; 0.71) | 0.29 | 0.48 (−0.28; 1.24) | 0.22 | −0.03 (−0.61; 0.56) | 0.93 |
| Calcium (mg/day/year) | −3.99 (−5.90; −2.08) | <0.001 | −0.60 (−3.45; 2.25) | 0.68 | −6.23 (−8.81; −3.66) | <0.001 |
| Iron (mg/day/year) | −0.01 (−0.01; 0.00) | 0.12 | −0.02 (−0.03; 0.00) | 0.01 | 0.00 (−0.01; 0.01) | 0.53 |
| Vitamin A (μg RAE/day) | −3.46 (−5.67; −1.25) | 0.002 | −3.54 (−6.88; −0.19) | 0.04 | −3.89 (−6.84; −0.93) | 0.01 |
| Retinol (µg/day/year) | −6.78 (−8.85; −4.71) | <0.001 | −6.22 (−9.33; −3.10) | <0.001 | −7.06 (−9.83; −4.29) | <0.001 |
| Carotene (µg/day/year) | 39.9 (30.0; 49.7) | <0.001 | 32.2 (15.6; 48.8) | <0.001 | 38.1 (25.7; 50.5) | <0.001 |
| Vitamin D (µg/day/year) | 0.01 (0.001; 0.02) | 0.04 | 0.01 (−0.01; 0.02) | 0.35 | 0.00 (−0.01; 0.01) | 0.94 |
| Alcohol (g/day/year) | −0.35 (−0.43; −0.27) | <0.001 | −0.31 (−0.41; −0.20) | <0.001 | −0.32 (−0.43; −0.21) | <0.001 |

**Table S3.** *Cont.*

|  | **All** | ***p*-Value** | **High** | ***p*-Value** | **Low** | ***p*-Value** |
| --- | --- | --- | --- | --- | --- | --- |
| **(b): Women** | | | | | | |
| **Sample Size** | **9009** |  | **3082** |  | **5927** |  |
| Total energy (Kcal/day/year) | −5.5 (−7.7; −3.3) | <0.001 | −7.7 (−11.3; −4.1) | <0.001 | −4.7 (−7.5; −2.0) | 0.001 |
| Macronutrients (g/day/year) |  |  |  |  |  |  |
| Proteins, total | 0.04 (−0.01; 0.09) | 0.15 | −0.03 (−0.12; 0.06) | 0.49 | 0.08 (0.01; 0.15) | 0.02 |
| Vegetal | 0.02 (0.001; 0.04) | 0.03 | 0.03 (−0.01; 0.07) | 0.10 | 0.01 (−0.01; 0.04) | 0.36 |
| Animal | 0.02 (−0.04; 0.08) | 0.56 | −0.06 (−0.16; 0.04) | 0.24 | 0.07 (−0.01; 0.14) | 0.09 |
| Carbohydrates | 0.21 (0.05; 0.37) | 0.008 | 0.32 (0.06; 0.58) | 0.02 | 0.17 (−0.03; 0.36) | 0.09 |
| Mono/disaccharides | 0.28 (0.14; 0.42) | <0.001 | 0.26 (0.03; 0.49) | 0.03 | 0.31 (0.13; 0.49) | 0.001 |
| Polysaccharides | −0.07 (−0.21; 0.07) | 0.36 | 0.07 (−0.16; 0.30) | 0.56 | −0.14 (−0.32; 0.04) | 0.12 |
| Fibres (g/day/year) |  |  |  |  |  |  |
| Total | 0.02 (−0.005; 0.04) | 0.12 | 0.01 (−0.03; 0.05) | 0.64 | 0.02 (−0.01; 0.05) | 0.23 |
| Cereal | −0.04 (−0.06; −0.02) | <0.001 | −0.04 (−0.07; −0.01) | 0.01 | −0.04 (−0.06; −0.02) | <0.001 |
| Fruits and vegetables | 0.06 (0.05; 0.08) | <0.001 | 0.06 (0.02; 0.09) | <0.001 | 0.07 (0.04; 0.09) | <0.001 |
| Total fat (g/day/year) | −0.01 (−0.06; 0.05) | 0.83 | 0.03 (−0.07; 0.12) | 0.58 | −0.02 (−0.09; 0.05) | 0.50 |
| SFA | −0.08 (−0.10; −0.05) | <0.001 | −0.05 (−0.10; −0.01) | 0.02 | −0.09 (−0.12; −0.05) | <0.001 |
| MUFA | 0.13 (0.10; 0.16) | <0.001 | 0.14 (0.09; 0.19) | <0.001 | 0.12 (0.09; 0.16) | <0.001 |
| PUFA | −0.08 (−0.09; −0.06) | <0.001 | −0.07 (−0.10; −0.05) | <0.001 | −0.08 (−0.09; −0.06) | <0.001 |
| Micronutrients |  |  |  |  |  |  |
| Cholesterol (mg/day/year) | 0.25 (−0.16; 0.65) | 0.27 | 0.33 (−0.40; 1.06) | 0.38 | 0.15 (−0.34; 0.64) | 0.56 |
| Calcium (mg/day/year) | −3.66 (−5.17; −2.15) | <0.001 | −3.21 (−5.67; −0.75) | 0.01 | −3.64 (−5.57; −1.71) | <0.001 |
| Iron (mg/day/year) | −0.01 (−0.02; −0.01) | <0.001 | −0.03 (−0.04; −0.02) | <0.001 | −0.01 (−0.02; 0.00) | 0.12 |
| Vitamin A (μg RAE/day) | −4.30 (−6.46; −2.15) | <0.001 | −4.00 (−7.49; −0.51) | 0.03 | −4.37 (−7.12; −1.61) | 0.002 |
| Retinol (µg/day/year) | −8.95 (−10.9; −7.02) | <0.001 | −8.41 (−11.40; −5.44) | <0.001 | −8.72 (−11.20; −6.21) | <0.001 |
| Carotene (µg/day/year) | 55.7 (43.2; 68.3) | <0.001 | 52.9 (30.0; 75.8) | <0.001 | 52.3 (37.2; 67.3) | <0.001 |
| Vitamin D (µg/day/year) | 0.01 (0.00; 0.01) | 0.053 | 0.01 (−0.01; 0.02) | 0.40 | 0.00 (0.00; 0.01) | 0.35 |
| Alcohol (g/day/year) | −0.13 (−0.18; −0.09) | <0.001 | −0.20 (−0.27; −0.12) | <0.001 | −0.11 (−0.17; −0.05) | <0.001 |

SFA, saturated fatty acids; MUFA, monounsaturated fatty acids; PUFA, poly-unsaturated fatty acids; RAE, retinol activity equivalent. Results are expressed as yearly change and (95% confidence interval). Statistical analysis by linear regression using yearly data adjusting for total energy intake (for nutrients).

**Table S4.** Age-adjusted differences in total energy and nutrient intake between educational levels in the “Bus Santé” study, Geneva, Switzerland, 1993–2012, according to gender and survey period.

|  | **Men** | | | | **Women** | | | |
| --- | --- | --- | --- | --- | --- | --- | --- | --- |
|  | **1993–1999** | ***p*-Value** | **2006–2012** | ***p*-Value** | **1993–1999** | ***p*-Value** | **2006–2012** | ***p*-Value** |
| **Sample Size** | **3257** |  | **2138** |  | **3665** |  | **2169** |  |
| Total energy (Kcal/day) | 22 (−27; 70) | 0.38 | 68 (12; 124) | 0.02 | −54 (−95; −13) | 0.01 | −30 (−80; 19) | 0.23 |
| Macronutrients (g/day) |  |  |  |  |  |  |  |  |
| Proteins, total | −0.30 (−1.43; 0.84) | 0.61 | −1.16 (−2.60; 0.29) | 0.12 | −0.44 (−1.43; 0.55) | 0.38 | 0.67 (−0.64; 1.98) | 0.31 |
| Vegetal | −0.82 (−1.30; −0.35) | <0.001 | −0.42 (−0.98; 0.13) | 0.14 | −0.62 (−1.03; −0.21) | 0.003 | −0.59 (−1.07; −0.10) | 0.02 |
| Animal | 0.54 (−0.76; 1.83) | 0.42 | −0.74 (−2.38; 0.90) | 0.38 | 0.18 (−0.94; 1.31) | 0.75 | 1.25 (−0.24; 2.74) | 0.10 |
| Carbohydrates, total | −2.69 (−6.26; 0.88) | 0.14 | 0.07 (−3.96; 4.10) | 0.97 | 0.77 (−2.18; 3.72) | 0.61 | 1.44 (−1.97; 4.85) | 0.41 |
| Mono/disaccharides | −0.87 (−3.75; 2.01) | 0.55 | −1.07 (−4.41; 2.27) | 0.53 | −0.11 (−2.74; 2.52) | 0.93 | 1.45 (−1.76; 4.67) | 0.37 |
| Polysaccharides | −2.05 (−5.19; 1.08) | 0.20 | 1.06 (−2.50; 4.62) | 0.56 | 0.77 (−1.89; 3.43) | 0.57 | −0.11 (−3.21; 3.00) | 0.95 |
| Fibres |  |  |  |  |  |  |  |  |
| Total | −1.25 (−1.75; −0.76) | <0.001 | −0.94 (−1.53; −0.35) | 0.002 | −1.09 (−1.55; −0.63) | <0.001 | −0.75 (−1.30; −0.19) | 0.008 |
| Cereal | −0.77 (−1.17; −0.37) | <0.001 | −0.42 (−0.86; 0.03) | 0.07 | −0.63 (−0.97; −0.29) | <0.001 | −0.43 (−0.82; −0.03) | 0.03 |
| Fruits and vegetables | −0.44 (−0.76; −0.12) | 0.006 | −0.40 (−0.82; 0.02) | 0.06 | −0.45 (−0.79; −0.11) | 0.01 | −0.29 (−0.73; 0.15) | 0.20 |
| Total fat | −0.80 (−2.05; 0.46) | 0.21 | −1.59 (−2.98; −0.19) | 0.03 | 0.93 (−0.12; 1.98) | 0.08 | −0.63 (−1.85; 0.59) | 0.31 |
| SFA | 0.43 (−0.21; 1.07) | 0.18 | −0.76 (−1.45; −0.08) | 0.03 | 0.84 (0.32; 1.35) | 0.001 | 0.02 (−0.55; 0.59) | 0.94 |
| MUFA | −0.94 (−1.53; −0.35) | 0.002 | −0.83 (−1.55; −0.11) | 0.02 | −0.13 (−0.67; 0.41) | 0.64 | −0.81 (−1.51; −0.12) | 0.02 |
| PUFA | −0.07 (−0.40; 0.27) | 0.70 | 0.27 (−0.05; 0.60) | 0.10 | 0.28 (0.01; 0.55) | 0.04 | 0.26 (−0.01; 0.52) § | 0.06 |
| Micronutrients |  |  |  |  |  |  |  |  |
| Cholesterol (mg/day) | −4.75 (−13.2; 3.71) | 0.27 | −8.77 (−19.0; 1.45) | 0.09 | −0.96 (−8.55; 6.64) | 0.81 | −4.9 (−15.4; 5.61) | 0.36 |
| Calcium (mg/day) | 33.5 (−3.8; 70.8) | 0.08 | −45.6 (−84.5; −6.8) | 0.02 | 11.9 (−17.3; 41.1) | 0.42 | −13.2 (−45.5; 19.1) | 0.42 |
| Iron (mg/day) | −0.31 (−0.46; −0.16) | <0.001 | −0.14 (−0.32; 0.03) | 0.10 | −0.41 (−0.55; −0.28) | <0.001 | −0.06 (−0.22; 0.09) | 0.42 |
| Vitamin A (μg RAE/day) | −20.0 (−62.4; 22.5) | 0.36 | −10.4 (−58.7; 37.9) | 0.67 | −2.8 (−45.3; 39.7) | 0.90 | −39.6 (−83.5; 4.2) | 0.08 |
| Retinol (µg/day) | 12.1 (−28.1; 52.2) | 0.56 | 15.2 (−29.9; 60.3) | 0.51 | 35.1 (−3.7; 73.9) | 0.82 | −4.2 (−41.8; 33.3) | 0.26 |
| Carotene (µg/day) | −384 (−559; −209) | <0.001 | −307 (−537; −77) | 0.009 | −454 (−675; −234) | <0.001 | −424 (−739; −109) | 0.008 |
| Vitamin D (µg/day) | −0.45 (−0.59; −0.30) | <0.001 | −0.54 (−0.72; −0.36) | <0.001 | −0.34 (−0.47; −0.21) | <0.001 | −0.31 (−0.48; −0.15) | <0.001 |
| Alcohol (g/day) | 2.84 (1.28; 4.40) | <0.001 | 2.62 (1.10; 4.14) | <0.001 | −1.36 (−2.32; −0.40) | 0.005 | −0.42 (−1.28; 0.44) | 0.34 |

SFA, saturated fatty acids; MUFA, monounsaturated fatty acids; PUFA, poly-unsaturated fatty acids; RAE, retinol activity equivalent. Results are expressed as adjusted difference and (95% confidence interval) between low and high educational levels for survey periods 1993–1999 and 2006–2012. Statistical analysis by analysis of variance adjusting for age and total energy intake (for nutrients).

**Table S5.** Trends in total energy and nutrient intakes in the “Bus Santé” study, Geneva, Switzerland, 1993–2012, overall and according to high or low educational level. All participants included irrespective of age or energy intake, or missing data for body mass index.

|  | **All** | ***p*-Value** | **High** | ***p*-Value** | **Low** | ***p*-Value** | ***p*-Value §** | ***p*-Value §§** |
| --- | --- | --- | --- | --- | --- | --- | --- | --- |
| **(a): Men** | | | | | | | | |
| **Sample Size** | **8884** |  | **3263** |  | **5621** |  |  |  |
| Total energy (Kcal/day/year) | −8.6 (−11.5; −5.7) | <0.001 | −11.3 (−15.9; −6.7) | <0.001 | −6.5 (−10.3; −2.7) | 0.001 | 0.09 | 0.10 |
| Macronutrients (g/day/year) |  |  |  |  |  |  |  |  |
| Proteins, total | 0.13 (0.07; 0.19) | <0.001 | 0.15 (0.05; 0.24) | 0.002 | 0.11 (0.03; 0.20) | 0.007 | 0.66 | 0.63 |
| Vegetal | 0.02 (0.00; 0.05) | 0.08 | 0.01 (−0.03; 0.05) | 0.77 | 0.02 (−0.01; 0.05) | 0.18 | 0.26 | 0.43 |
| Animal | 0.11 (0.04; 0.18) | 0.003 | 0.14 (0.03; 0.26) | 0.10 | 0.09 (0.00; 0.19) | 0.05 | 0.43 | 0.48 |
| Carbohydrates | 0.31 (0.13; 0.50) | 0.001 | 0.18 (−0.11; 0.47) | 0.23 | 0.41 (0.17; 0.66) | 0.001 | 0.09 | 0.15 |
| Mono/disaccharides | 0.36 (0.21; 0.51) | <0.001 | 0.31 (0.08; 0.54) | 0.009 | 0.12 (−0.19; 0.43) | <0.001 | 0.42 | 0.45 |
| Polysaccharides | −0.04 (−0.20; 0.12) | 0.62 | −0.13 (−0.39; 0.12) | 0.31 | 0.00 (−0.21; 0.21) | 0.98 | 0.21 | 0.35 |
| Fibres (g/day/year) |  |  |  |  |  |  |  |  |
| Total | 0.03 (0.01; 0.06) | 0.01 | 0.01 (−0.03; 0.05) | 0.58 | 0.03 (0.01; 0.07) | 0.05 | 0.21 | 0.40 |
| Cereal | −0.03 (−0.05; −0.01) | 0.01 | −0.04 (−0.08; −0.01) | 0.01 | −0.02 (−0.05; 0.003) | 0.08 | 0.26 | 0.34 |
| Fruits and vegetables | 0.06 (0.05; 0.08) | <0.001 | 0.06 (0.03; 0.09) | <0.001 | 0.06 (0.04; 0.09) | <0.001 | 0.54 | 0.95 |
| Total fat (g/day/year) | 0.04 (−0.03; 0.10) | 0.26 | 0.05 (−0.05; 0.15) | 0.33 | 0.01 (−0.07; 0.10) | 0.77 | 0.22 | 0.59 |
| SFA | −0.08 (−0.11; −0.05) | <0.001 | −0.05 (−0.10; 0.00) | 0.06 | −0.11 (−0.15; −0.06) | <0.001 | 0.007 | 0.08 |
| MUFA | 0.14 (0.11; 0.18) | <0.001 | 0.13 (0.08; 0.18) | <0.001 | 0.14 (0.10; 0.18) | <0.001 | 0.88 | 0.82 |
| PUFA | −0.05 (−0.07; −0.04) | <0.001 | −0.06 (−0.09; −0.04) | <0.001 | −0.05 (−0.07; −0.02) | <0.001 | 0.35 | 0.38 |
| Micronutrients |  |  |  |  |  |  |  |  |
| Cholesterol (mg/day/year) | 0.41 (−0.06; 0.88) | 0.09 | 0.70 (−0.09; 1.49) | 0.08 | 0.17 (−0.42; 0.76) | 0.57 | 0.43 | 0.47 |
| Calcium (mg/day/year) | −3.65 (−5.52; −1.78) | <0.001 | −1.07 (−3.86; 1.71) | 0.45 | −5.51 (−8.03; −3.00) | <0.001 | 0.003 | 0.02 |
| Iron (mg/day/year) | −0.01 (−0.01; 0.00) | 0.22 | −0.01 (−0.02; −0.002) | 0.04 | 0.00 (−0.01; 0.01) | 0.54 | 0.18 | 0.28 |
| Vitamin A (μg RAE/day) | −3.87 (−6.09; −1.64) | 0.001 | −4.14 (−7.59; −0.69) | 0.02 | −3.99 (−6.93; −1.04) | 0.008 | 0.98 | 0.98 |
| Retinol (µg/day/year) | −7.21 (−9.29; −5.13) | <0.001 | −7.05 (−10.3; −3.83) | <0.001 | −7.29 (−10.0; −4.54) | <0.001 | 0.78 | 0.86 |
| Carotene (µg/day/year) | 40.2 (30.4; 50.0) | <0.001 | 34.9 (18.6; 51.2) | <0.001 | 39.6 (27.2; 52.0) | <0.001 | 0.53 | 0.60 |
| Vitamin D (µg/day/year) | 0.01 (0.00; 0.02) | 0.04 | 0.01 (−0.01; 0.02) | 0.18 | 0.00 (−0.01; 0.01) | 0.74 | 0.48 | 0.47 |
| Alcohol (g/day/year) | −0.3 (−0.38; −0.23) | <0.001 | −0.25 (−0.35; −0.15) | <0.001 | −0.32 (−0.43; −0.21) | <0.001 | 0.36 | 0.22 |

**Table S5.** *Cont.*

|  | **All** | ***p*-Value** | **High** | ***p*-Value** | **Low** | ***p*-Value** | ***p*-Value §** | ***p*-Value §§** |
| --- | --- | --- | --- | --- | --- | --- | --- | --- |
| **(b): Women** | | | | | | | | |
| **Sample Size** | **9779** |  | **3336** |  | **6443** |  |  |  |
| Total energy (Kcal/day/year) | −5.9 (−8.2; −3.6) | <0.001 | −8.9 (−12.6; −5.2) | <0.001 | −4.8 (−7.7; −1.8) | 0.002 | 0.09 | 0.08 |
| Macronutrients (g/day/year) |  |  |  |  |  |  |  |  |
| Proteins, total | 0.02 (−0.03; 0.07) | 0.43 | −0.04 (−0.12; 0.05) | 0.41 | 0.05 (−0.02; 0.11) | 0.14 | 0.13 | 0.51 |
| Vegetal | 0.01 (−0.02; 0.03) | 0.61 | 0.02 (−0.02; 0.05) | 0.31 | −0.01 (−0.03; 0.02) | 0.67 | 0.41 | 0.41 |
| Animal | 0.02 (−0.04; 0.07) | 0.58 | −0.05 (−0.15; 0.04) | 0.29 | 0.05 (−0.02; 0.13) | 0.14 | 0.10 | 0.12 |
| Carbohydrates | 0.14 (0.00; 0.29) | 0.05 | 0.23 (−0.02; 0.47) | 0.07 | 0.12 (−0.06; 0.31) | 0.19 | 0.53 | 0.57 |
| Mono/disaccharides | 0.27 (0.13; 0.40) | <0.001 | 0.18 (−0.03; 0.40) | 0.10 | 0.33 (0.16; 0.50) | <0.001 | 0.43 | 0.36 |
| Polysaccharides | −0.12 (−0.25; 0.01) | 0.08 | 0.05 (−0.17; 0.27) | 0.66 | −0.20 (−0.37; −0.04) | 0.02 | 0.14 | 0.12 |
| Fibres (g/day/year) |  |  |  |  |  |  |  |  |
| Total | 0.00 (−0.02; 0.02) | 0.96 | −0.01 (−0.04; 0.03) | 0.77 | 0.00 (−0.03; 0.03) | 0.94 | 0.67 | 0.66 |
| Cereal | −0.05 (−0.06; −0.03) | <0.001 | −0.05 (−0.08; −0.02) | 0.001 | −0.05 (−0.07; −0.03) | <0.001 | 0.93 | 0.89 |
| Fruits and vegetables | 0.05 (0.04; 0.07) | <0.001 | 0.05 (0.02; 0.08) | <0.001 | 0.05 (0.03; 0.08) | <0.001 | 0.58 | 0.67 |
| Total fat (g/day/year) | 0.03 (−0.02; 0.08) | 0.24 | 0.06 (−0.03; 0.14) | 0.20 | 0.02 (−0.05; 0.08) | 0.57 | 0.38 | 0.49 |
| SFA | −0.06 (−0.08; −0.03) | <0.001 | −0.04 (−0.08; 0.00) | 0.08 | −0.06 (−0.09; −0.03) | <0.001 | 0.14 | 0.27 |
| MUFA | 0.14 (0.11; 0.17) | <0.001 | 0.15 (0.10; 0.19) | <0.001 | 0.14 (0.10; 0.17) | <0.001 | 0.80 | 0.83 |
| PUFA | −0.07 (−0.08; −0.06) | <0.001 | −0.07 (−0.09; −0.05) | <0.001 | −0.07 (−0.08; −0.05) | <0.001 | 0.96 | 0.93 |
| Micronutrients |  |  |  |  |  |  |  |  |
| Cholesterol (mg/day/year) | 0.33 (−0.06; 0.72) | 0.10 | 0.44 (−0.26; 1.14) | 0.22 | 0.26 (−0.21; 0.73) | 0.28 | 0.74 | 0.66 |
| Calcium (mg/day/year) | −3.62 (−5.03; −2.21) | <0.001 | −3.44 (−5.79; −1.10) | 0.004 | −3.83 (−5.60; −2.05) | <0.001 | 0.45 | 0.72 |
| Iron (mg/day/year) | −0.02 (−0.03; −0.01) | <0.001 | −0.03 (−0.04; −0.02) | <0.001 | −0.01 (−0.02; −0.001) | 0.004 | 0.002 | 0.004 |
| Vitamin A (μg RAE/day) | −4.29 (−6.41; −2.18) | <0.001 | −3.42 (−6.72; −0.13) | 0.04 | −4.79 (−7.53; −2.05) | 0.001 | 0.51 | 0.50 |
| Retinol (µg/day/year) | −8.37 (−10.25; −6.49) | <0.001 | −7.45 (−10.3; −4.63) | <0.001 | −8.63 (−11.1; −6.15) | <0.001 | 0.46 | 0.45 |
| Carotene (µg/day/year) | 48.9 (37.0; 60.7) | <0.001 | 48.3 (26.7; 69.9) | <0.001 | 46.1 (31.9; 60.3) | <0.001 | 0.99 | 0.99 |
| Vitamin D (µg/day/year) | 0.01 (0.00; 0.02) | 0.005 | 0.01 (−0.004; 0.02) | 0.23 | 0.01 (0.001; 0.02) | 0.05 | 0.80 | 0.82 |
| Alcohol (g/day/year) | −0.13 (−0.17; −0.09) | <0.001 | −0.18 (−0.25; −0.11) | <0.001 | −0.12 (−0.17; −0.07) | <0.001 | 0.14 | 0.25 |

SFA, saturated fatty acids; MUFA, monounsaturated fatty acids; PUFA, poly-unsaturated fatty acids; RAE, retinol activity equivalent. Results are expressed as yearly change and (95% confidence interval). § *p*-value for the interaction between survey year and education, unadjusted or adjusting for total energy (for nutrients); §§ *p*-value for the interaction between survey year and education, adjusting for age, marital status, country of birth (dichotomized into Switzerland yes/no), smoking and total energy (for nutrients). Statistical analysis by linear regression using yearly data adjusting for age, marital status, country of birth, smoking and total energy intake (for nutrients).

**Table S6.** Multivariable-adjusted differences in total energy and nutrient intake between educational levels in the “Bus Santé” study, Geneva, Switzerland, 1993–2012, according to gender and survey period. All participants included irrespective of age or energy intake, or missing data for body mass index.

|  | **Men** | | | | **Women** | | | |
| --- | --- | --- | --- | --- | --- | --- | --- | --- |
|  | **1993–1999** | ***p*−Value** | **2006–2012** | ***p*-Value** | **1993–1999** | ***p*-Value** | **2006–2012** | ***p*-Value** |
| **Sample Size** | **3461** |  | **2365** |  | **3889** |  | **2473** |  |
| Total energy (Kcal/day) | 30 (−28; 88) | 0.32 | 67 (3; 132) | 0.04 | −55 (−101; −10) | 0.02 | −17 (−70; 37) | 0.55 |
| Macronutrients (g/day) |  |  |  |  |  |  |  |  |
| Proteins, total | −0.26 (−1.44; 0.93) | 0.67 | −0.88 (−2.32; 0.56) | 0.23 | −0.11 (−1.09; 0.87) | 0.82 | 0.5 (−0.74; 1.74) | 0.43 |
| Vegetal | −0.46 (−0.94; 0.02) | 0.06 | −0.37 (−0.92; 0.18) | 0.18 | −0.46 (−0.86; −0.06) | 0.02 | −0.61 (−1.07; −0.16) | 0.008 |
| Animal | 0.21 (−1.13; 1.55) | 0.76 | −0.51 (−2.13; 1.11) | 0.54 | 0.35 (−0.76; 1.47) | 0.54 | 1.11 (−0.29; 2.52) | 0.12 |
| Carbohydrates, total | 0.14 (−3.49; 3.77) | 0.94 | 1.73 (−2.23; 5.70) | 0.39 | 1.78 (−1.09; 4.64) | 0.22 | 2.45 (−0.79; 5.70) | 0.14 |
| Mono/disaccharides | 0.26 (−2.70; 3.22) | 0.87 | 0.32 (−3.02; 3.66) | 0.85 | 0.40 (−2.20; 2.99) | 0.76 | 2.92 (−0.14; 5.98) | 0.06 |
| Polysaccharides | −0.25 (−3.39; 2.90) | 0.88 | 1.30 (−2.22; 4.81) | 0.47 | 1.29 (−1.32; 3.89) | 0.33 | −0.54 (−3.44; 2.37) | 0.72 |
| Fibres |  |  |  |  |  |  |  |  |
| Total | −0.70 (−1.20; −0.20) | 0.006 | −0.72 (−1.29; −0.14) | 0.01 | −0.80 (−1.25; −0.36) | <0.001 | −0.59 (−1.11; −0.07) | 0.03 |
| Cereal | −0.51 (−0.92; −0.11) | 0.01 | −0.38 (−0.81; 0.06) | 0.09 | −0.60 (−0.93; −0.26) | <0.001 | −0.49 (−0.86; −0.12) | 0.01 |
| Fruits and vegetables | −0.12 (−0.44; 0.21) | 0.48 | −0.30 (−0.70; 0.11) | 0.15 | −0.18 (−0.52; 0.16) | 0.30 | −0.09 (−0.50; 0.33) | 0.68 |
| Total fat | −1.73 (−3.02; −0.44) | 0.009 | −1.74 (−3.12; −0.36) | 0.01 | 0.53 (−0.48; 1.55) | 0.30 | −0.89 (−2.07; 0.29) | 0.14 |
| SFA | −0.24 (−0.89; 0.41) | 0.47 | −0.86 (−1.53; −0.18) | 0.01 | 0.51 (0.01; 1.00) | 0.05 | −0.24 (−0.78; 0.31) | 0.39 |
| MUFA | −1.13 (−1.75; −0.51) | <0.001 | −0.92 (−1.63; −0.22) | 0.01 | −0.21 (−0.73; 0.32) | 0.43 | −0.86 (−1.53; −0.19) | 0.01 |
| PUFA | −0.13 (−0.46; 0.20) | 0.45 | 0.29 (−0.05; 0.62) | 0.10 | 0.30 (0.04; 0.56) | 0.02 | 0.30 (0.04; 0.55) | 0.02 |
| Micronutrients |  |  |  |  |  |  |  |  |
| Cholesterol (mg/day) | −9.94 (−18.71; −1.18) | 0.03 | −10.71 (−21.5; 0.04) | 0.05 | 1.33 (−6.30; 8.95) | 0.73 | −3.09 (−13.01; 6.82) | 0.54 |
| Calcium (mg/day) | 25.2 (−12.7; 63.0) | 0.19 | −50.4 (−88.2; −12.6) | 0.009 | −5.9 (−34.4; 22.6) | 0.68 | −27.8 (−58.1; 2.6) | 0.07 |
| Iron (mg/day) | −0.28 (−0.44; −0.12) | <0.001 | −0.13 (−0.30; 0.04) | 0.14 | −0.36 (−0.49; −0.22) | <0.001 | −0.06 (−0.20; 0.09) | 0.45 |
| Vitamin A (μg RAE/day) | −40.2 (−85.6; 5.2) | 0.08 | −11.6 (−57.5; 34.4) | 0.62 | −1.5 (−44.8; 41.9) | 0.95 | −26.2 (−73.2; 20.8) | 0.27 |
| Retinol (µg/day) | −14.1 (−57.0; 28.9) | 0.52 | 8.2 (−34.1; 50.4) | 0.70 | 25.0 (−147.0; 64.6) | 0.22 | −1.4 (−41.6; 38.7) | 0.94 |
| Carotene (µg/day) | −313 (−491; −135) | <0.001 | −237 (−469; −5) | 0.05 | −317 (−535; −99) | 0.004 | −297 (−589; −5) | 0.05 |
| Vitamin D (µg/day) | −0.45 (−0.59; −0.30) | <0.001 | −0.51 (−0.69; −0.32) | <0.001 | −0.3 (−0.43; −0.18) | <0.001 | −0.21 (−0.40; −0.03) | 0.02 |
| Alcohol | 2.35 (0.80; 3.89) | 0.003 | 1.73 (0.26; 3.20) | 0.02 | −1.62 (−2.53; −0.71) | <0.001 | −0.57 (−1.37; 0.23) | 0.16 |

SFA, saturated fatty acids; MUFA, monounsaturated fatty acids; PUFA, poly-unsaturated fatty acids; RAE, retinol activity equivalent. Results are expressed as multivariable adjusted differences (low minus high) and (95% confidence interval) in nutrient intake between educational categories. Statistical analysis by analysis of variance adjusting for age, country of birth, marital status (dichotomized into Switzerland yes/no), smoking status and total energy intake (for nutrients).

**Table S7.** Comparison between participants included and under-reporters.

|  | **Included** | | **Under-Reporters** | | ***p*-Value** |
| --- | --- | --- | --- | --- | --- |
| ***N*** | **17,263** | | **471** | |  |
| Age (years) | 52.0 | (10.6) | 51.9 | (10.6) | 0.82 |
| BMI (kg/m^2^) | 24.7 | (4.0) | 24.6 | (4.4) | 0.64 |
| Gender |  |  |  |  |  |
| Male | 8254 | (47.8) | 91 | (19.3) | <0.001 |
| Female | 9009 | (52.2) | 380 | (80.7) |  |
| Education |  |  |  |  |  |
| High | 6088 | (35.3) | 122 | (26.6) | <0.001 |
| Low | 11,175 | (64.7) | 337 | (73.4) |  |
| Country of birth |  |  |  |  |  |
| Switzerland | 9498 | (55.0) | 217 | (46.1) | <0.001 |
| Other | 7765 | (45.0) | 254 | (53.9) |  |
| Marital status |  |  |  |  |  |
| Single | 1684 | (9.8) | 77 | (16.4) | <0.001 |
| Married | 12,675 | (73.4) | 245 | (52.0) |  |
| Divorced | 2283 | (13.2) | 119 | (25.3) |  |
| Widowed | 621 | (3.6) | 30 | (6.4) |  |
| Smoking status |  |  |  |  |  |
| Never | 7775 | (45.0) | 217 | (46.2) | 0.005 |
| Former | 5538 | (32.1) | 131 | (27.8) |  |
| Current | 3950 | (22.9) | 122 | (26.0) |  |

Results are expressed as number of participants (%) or as mean (standard deviation). BMI, body mass index. Statistical comparisons by chi-square or student’s *t*-test.
